# Supplementary material for: In Vitro Expansion of Vδ1+ T Cells from Cord Blood by Using Artificial Antigen-Presenting Cells and Anti-CD3 Antibody
Source: Vaccines (Basel). 2023 Feb 10;11(2):406. doi: 10.3390/vaccines11020406 (PMC9961230; doi:10.3390/vaccines11020406)

Supplementary Figure S1. Representative plot of distribution of Vδ1, Vδ2, and Vδ1-Vδ2- γδ T cells after negative isolation with magnetic beads from cord blood samples.

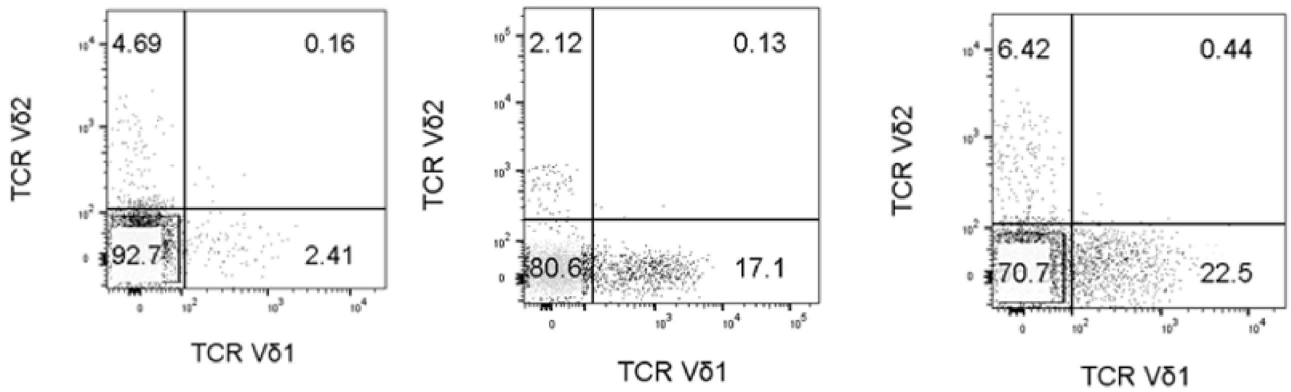

Supplementary Figure S2. Absolute cell number of Vd1+ T cells presence or absence of anti-CD3 antibody in culture for two weeks. The absolute cell numbers of 5 cordblood units increased. (n=5)

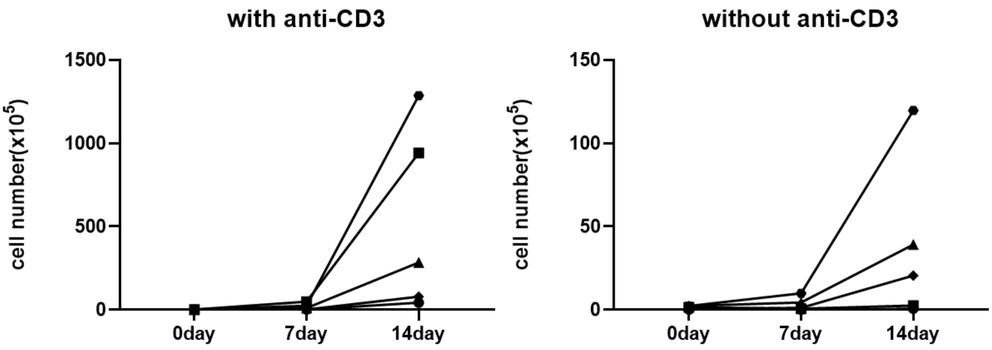

Supplementary Figure S3. Representative plot of distribution of Vδ1+, Vδ2+, and Vδ1-Vδ2- γδ T cells according to the presence or absence of anti-CD3 antibody in culture for two weeks.

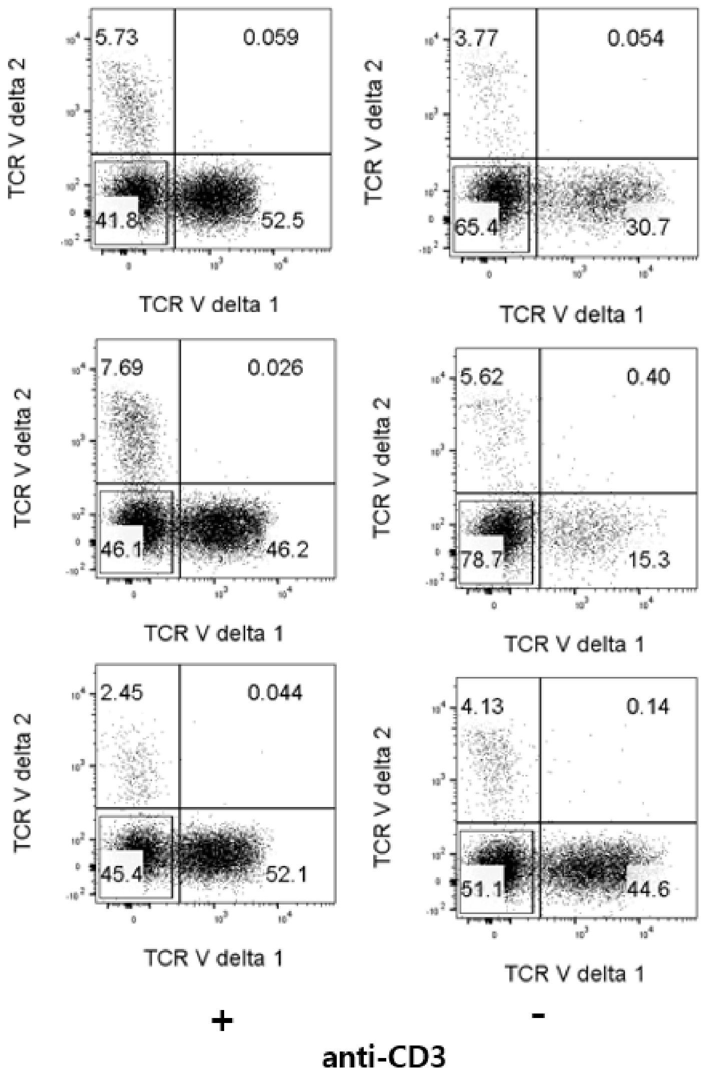

Supplementary Figure S4. Absolute cell number of Vδ1+ T cells with anti-CD3 antibody and aAPCs in culture for two weeks. The absolute cell numbers of 7 cordblood units increased. (n=7)

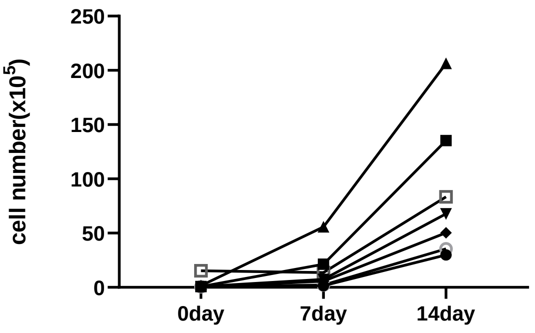

Supplementary Figure S5. Correlation analysis between the expression rate of each ligand.

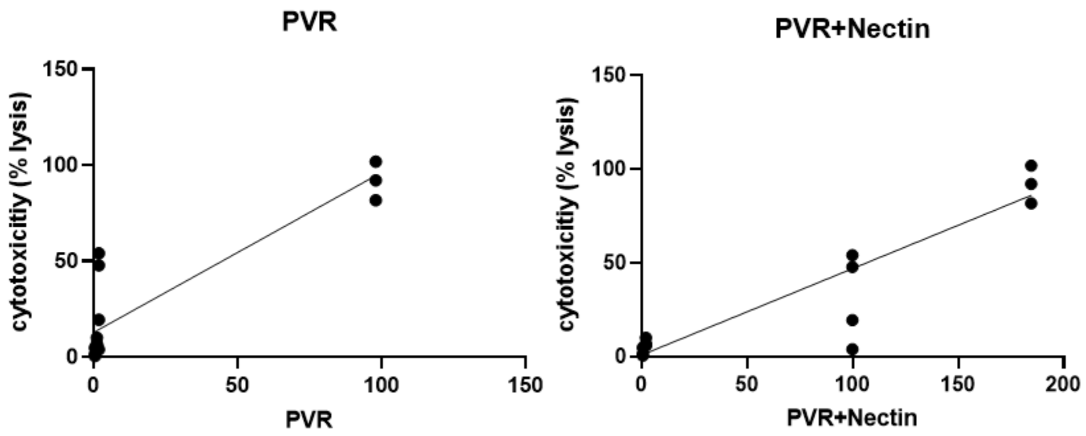

Supplementary Figure S6. Representative flow cytometry data of target cell ligands expression.

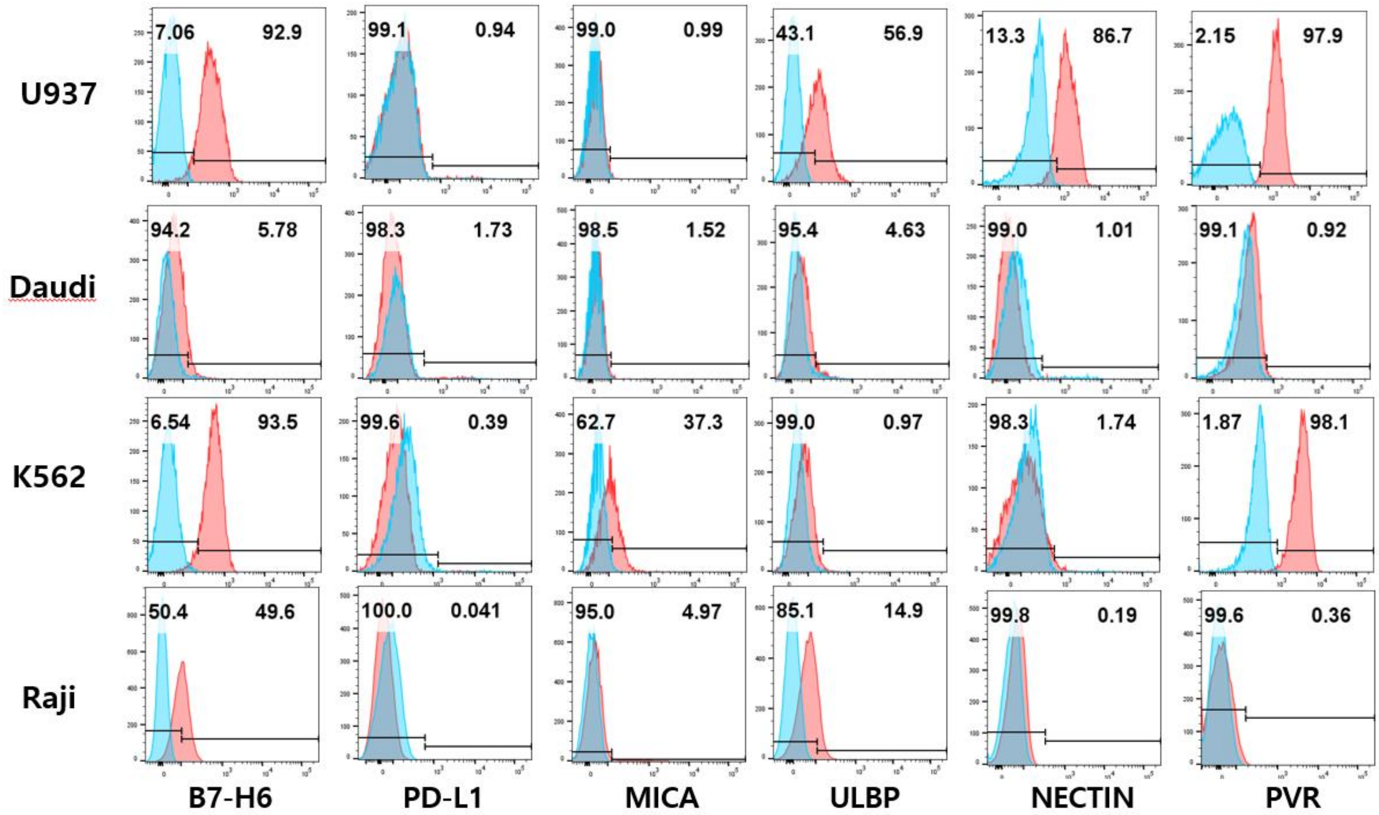

Supplementary Figure S7. Result of a blocking test for these receptors using antibodies against DNAM-1 and NKG2D expressed in Vd1+ T cells.

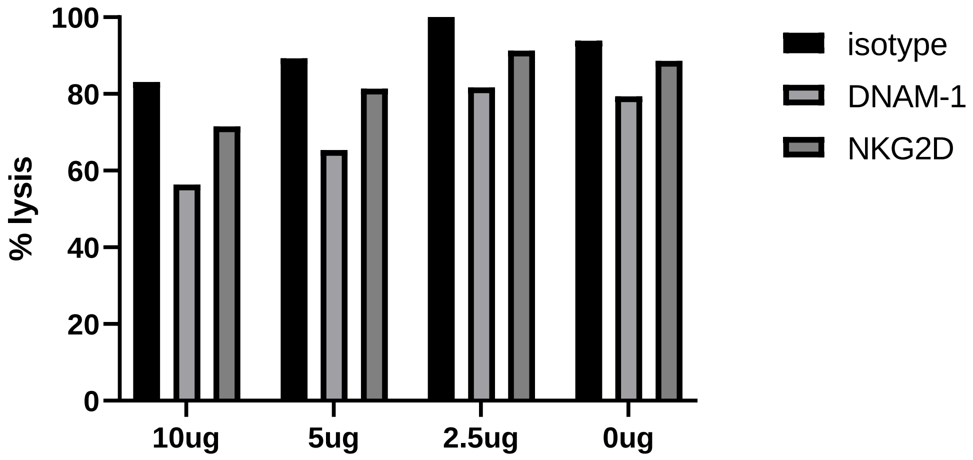

Supplement: Supplementary file 1 [file vaccines-11-00406-s001.zip › vaccines-2117177-supplementary.pdf]
